# Spatiotemporal variation of mosquito diversity (Diptera: Culicidae) at places with different land-use types within a neotropical montane cloud forest matrix

**DOI:** 10.1186/s13071-015-1086-9

**Published:** 2015-09-24

**Authors:** Carlos Antonio Abella-Medrano, Sergio Ibáñez-Bernal, Ian MacGregor-Fors, Diego Santiago-Alarcon

**Affiliations:** Instituto de Ecología A.C., Red Ambiente y Sustentabilidad, Carretera antigua a Coatepec 351 El Haya, Xalapa, 91070 Veracruz Mexico; Instituto de Ecología A.C., Red de Biología y Conservación de Vertebrados, Carretera antigua a Coatepec 351 El Haya, Xalapa, 91070 Veracruz Mexico

**Keywords:** Culicidae, Landscape, Land-use changes, Diversity, Anthropogenic effects, Urban ecology, Urban parasitology

## Abstract

**Background:**

Land-use change has led to a dramatic decrease in total forest cover, contributing to biodiversity loss and changes of ecosystems’ functions. Insect communities of medical importance can be favored by anthropogenic alterations, increasing the risk of novel zoonotic diseases. The response of mosquito (Diptera: Culicidae) abundance and richness to five land-use types (shade coffee plantation, cattle field, urban forest, peri-urban forest, well-preserved montane cloud forest) and three seasons (“dry”, “rainy” and “cold”) embedded in a neotropical montane cloud forest landscape was evaluated.

**Methods:**

Standardized collections were performed using 8 CDC miniature black-light traps, baited with CO2 throughout the year. Generalized additive mixed models were used to describe the seasonal and spatial trends of both species richness and abundance. Rank abundance curves and ANCOVAs were used to detect changes in the spatial and temporal structure of the mosquito assemblage. Two cluster analyses were conducted, using 1-βsim and the Morisita-Horn index to evaluate species composition shifts based on incidences and abundances.

**Results:**

A total of 2536 adult mosquitoes were collected, belonging to 9 genera and 10 species; the dominant species in the study were: *Aedes quadrivittatus*, *Wyeomyia adelpha*, *Wy. arthrostigma*, and *Culex restuans*. Highest richness was recorded in the dry season, whereas higher abundance was detected during the rainy season. The urban forest had the highest species richness (*n* = 7) when compared to all other sites. Species composition cluster analyses show that there is a high degree of similarity in species numbers across sites and seasons throughout the year. However, when considering the abundance of such species, the well-preserved montane cloud forest showed significantly higher abundance. Moreover, the urban forest is only 30 % similar to other sites in terms of species abundances, indicating a possible isolating role of the urban environment.

**Conclusion:**

Mosquito assemblage was differentially influenced by land-use change and seasonality, but at the same time the assemblage is rather homogeneous across the studied landscape, suggesting a high degree of spatial connectivity. Information generated in this study is potentially useful in the development of urban planning and surveillance programs focused mainly on mosquito species of medical and veterinary importance.

**Electronic supplementary material:**

The online version of this article (doi:10.1186/s13071-015-1086-9) contains supplementary material, which is available to authorized users.

## Background

Land-use change has led to a dramatic decrease of total forest cover and an increasing isolation among forest remnants, contributing to the current biodiversity loss, rising species extinction rates, and alterations of key functional properties of ecosystems [1–3]. For instance, changes in land-use and vegetation cover have caused a decline of bird diversity [4, 5] and of butterfly species in scrub forests, wetlands, and dry grassland [5, 6]. The abundance and species richness of some rodents depend on shrub vegetation that provides refuge and food, for this reason their diversity is lower in rangelands [7]. Thus, alterations of mosquito diversity by changes in land uses may disrupt the transmission dynamics of emerging and re-emerging zoonotic infectious diseases [8].

Studies on the effect of land-use changes on insect communities of medical importance (e.g., mosquitoes) are scarce, but in general show that anthropogenic alterations positively affect insect vector populations by creating favorable breeding conditions [9–11]. Land-use change has been repeatedly mentioned as an anthropogenic factor that exacerbates mosquito-borne diseases [12]. For example, clearing land for subsistence agriculture, dam construction for hydroelectric power or recreational use, often expands and creates new mosquito breeding habitats, facilitating the introduction of invasive species or allowing mosquito populations to rise, increasing the probability of disease transmission [9, 13, 14]. Moreover, the quantification of vector population responses to different degrees of anthropogenic disturbances is virtually nonexistent. Hence, how insect vector communities respond to alterations of the landscape is relevant to understanding the emergence of zoonotic pathogens [15, 16].

In this study we focused on understanding the responses of mosquito (Diptera: Culicidae) species richness and abundance, as well as their community structure and composition to different land use types. Our study was conducted in a region that was originally dominated by neotropical montane cloud forest. We expected to find a decline in mosquito diversity from preserved to urban forests due to homogenizing conditions (e.g., simplified habitat structure, more resources for anthropophilic mosquito species, breeding sites such as cans, tires) of cities that usually favor a few urban adapted species. Hence, higher mosquito diversity is expected in the well-preserved cloud forest due to its more natural and complex composition (i.e., well-defined vegetation layers) compared to simplified land types (e.g., shade coffee plantation, cattle field). We expected a similar community structure and composition among sites with similar vegetation structure (i.e., well-preserved cloud forest, peri-urban and urban forest) due to the high connectivity across the studied landscape. The study was performed in three defined seasons (“dry”, “rainy” and “cold”) of the year; higher mosquito richness and abundance during the rainy season was expected because environmental conditions (i.e., higher temperature and humidity) are more conducive for insect development.

## Methods

### Study area

**Fig. 1:**
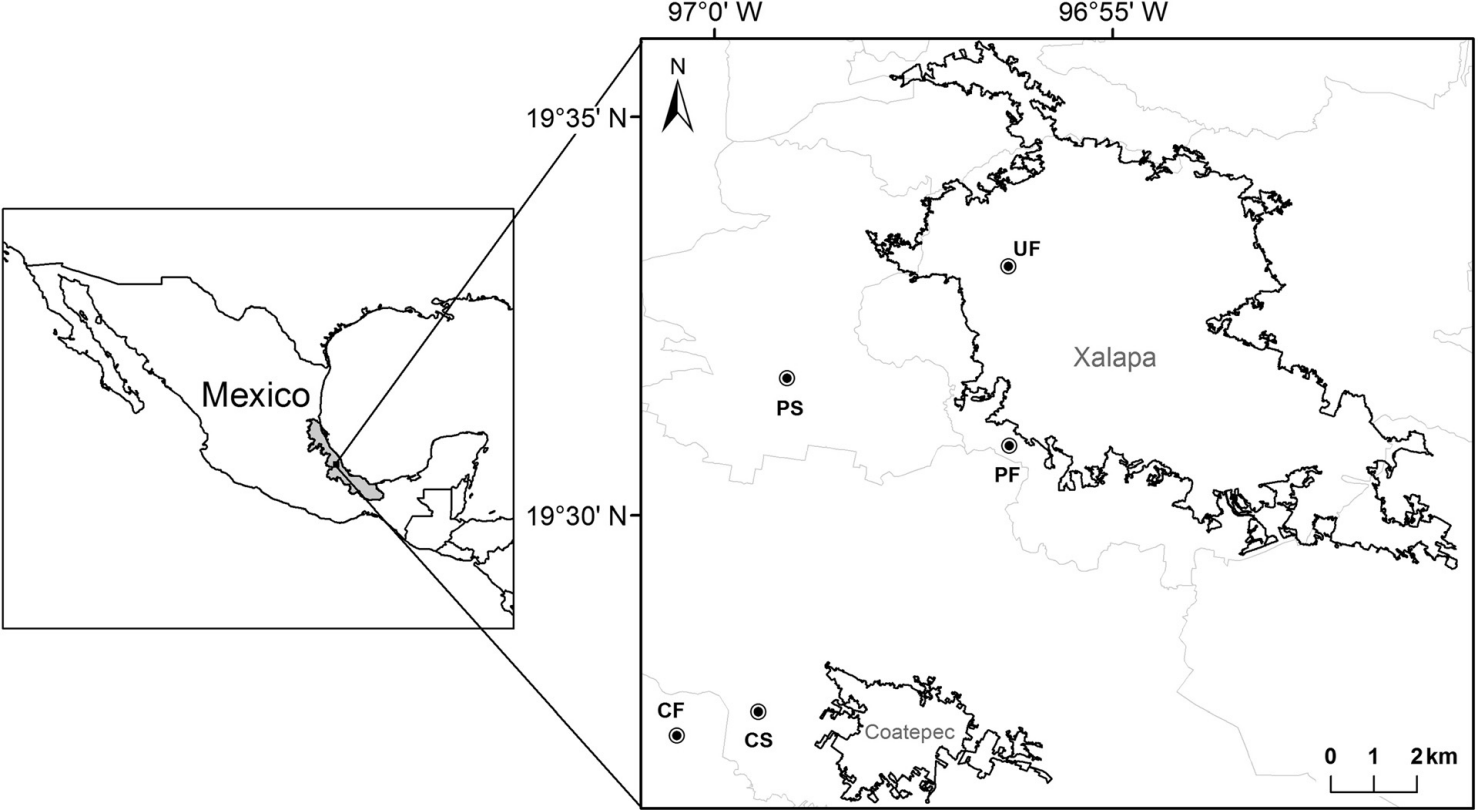
Study area and sampling sites (circles with central dot). Black lines indicate the limits of urban areas and gray lines the municipality borders. Sites: well-preserved montane cloud forest (CF), peri-urban forest (PF), urban forest (UF), shade coffee plantation (CS) and cattle field (PS)

The study was conducted in an area located in the central portion of the state of Veracruz, Mexico, where the original vegetation was montane cloud forest as described by Rzedowski (1978) (Fig. 1). The original vegetation has been heavily fragmented and there are a few isolated remnants within a matrix composed mostly of shade coffee plantations, cattle fields, and human settlements that are rapidly expanding [17–19]. According to the description of Contreras & Ornelas (1999), the well-preserved montane cloud forest present a mean annual rainfall on 1492 mm, with a minimum of 44.8 mm in December and a maximum of 273.4 mm in June. Mean annual temperature is 18 °C, with a maximum mean temperature of 20.4 °C in May and a minimum of 14.9 °C in January [20]. We sampled five sites, each with a different land-use type: 1) well-preserved montane cloud forest (CF), 2) urban forest (UF), 3) peri-urban forest (PF), 4) shade coffee plantation (CS), and 5) cattle field (PS) (Fig. 1).

The well-preserved montane cloud forest (CF) is located in the municipality of Xico, bordering Coatepec (19°27’15”N, 97°00’28”W; 1300–1500 masl). The floristic composition of this site is similar to that of other fragments of cloud forest in the region with the following dominant species: *Quercus affinis*, *Q. salicifolia*, *Q. leiophylla*, *Liquidambar styraciflua*, *Alchornea latifolia*, *Clethra mexicana*, *Myrsine coriaceae*, *Cinnamomum effusum*, *Vismia mexicana*, *Ilex* sp., *Eugenia* sp., *Ailanthus altissima*, *Turpinia insignis*, *Heliocarpus appendiculatus*, *Persea americana*, *Persea schiedeana* [21].

The urban forest (UF) (Park Molinos de San Roque) is a small urban natural protected area (15 ha) located in northwestern portion of the city of Xalapa (19°33’07”N, 96°56’18”W; 1427–1467 masl). This area retains much of its original cloud forest vegetation, with some small areas of second growth vegetation and an artificial swamp. Dominant species include: *Quercus xalapensis*, *Liquidambar macrophyla*, *Carpinus caroliniana*, *C. mexicana*, *Bocconia frutescens*, *Piper auritum*, *Ricinis communis*, *Melampodium divaricatum*, *Trichilia havanensis*, *Cynodon plectostachtyum*, *Typha dominguensis*, *Juncus* sp., and *Ciperus* sp. [22, 23].

The peri-urban forest (PF) (Ecologic Park Francisco Xavier Clavijero) is located in the southwestern border of the city of Xalapa, which is rapidly expanding (19°30’52”N, 96°56’12”W; 1344–1372 masl). Vegetation consists of second growth vegetation and abandoned coffee plantations. Some characteristic plants of this site are: *Quercus germana*, *Q. xalapensis*, *Platanus mexicanus*, *L. macrophyla*, *C. caroliniana*, *Cinnamomum effusum*, *Ocotea* sp., *Turpinia insignis*, *C. mexicana*, *Eugenia xalapensis*, *Lonchocarpus* sp., *Meliosma alba*, *Ilex tolucana*, *Oreopanax xalapensis*, and *Palicourea padifolia* [17, 18].

The shade coffee plantation (CS) is located in the municipality of Xico, bordering Coatepec (19°27’32”N, 96°59’26”W; 1210–1313 masl). The coffee plantation is characterized by the presence of some cloud forest elements and also by some exotic plant species: *Coffea* spp., *Inga jinicuil*, *P. americana*, *P. schiedeana*, *Heliocarpus appendiculatus*, *H. donnell-smithii*, *Rapanea myricoides*, *Trichilia havanensis*, *Leucaena leucocepaha*, *Malvaviscus arboreus*, *Palicourea padifolia*, *Piper nudum*, *Liquidambar styraciflua*, *Quercus* spp., and *Pinus patula* [21–24].

The cattle field (PS) is located in the municipality of San Andrés Tlalnelhuayocan (19°31’37”N, 96°59’7”W; 1460–1525 masl). As other cattle fields in the region, this site is open for grazing with scattered trees, shrubs, and herbs, surrounded by patches of cloud forest and second growth vegetation. Predominant scattered tree and shrubs species include: *P. mexicanus*, *Quercus* sp., *L. macrophyla*, *Acacia pennatula*, *Psidium guajava*, *Cnidoscolus* sp., *Piper* sp. (pers. obs.). The herbaceous stratum is dominated by native short grasses (e.g. *Axonopus compressus, Paspalum* spp.), an exotic tall grass (*Cynodon plectostachyus*), and other species (e.g. *Melampodium divaricatum, Borreria laevis, Hyptis atrorubens, Desmodium* sp., *Eupatorium* sp.) [25].

### Vector sampling

Sampling was conducted within a half-hectare square grid (100 × 50 m) at each site using eight CDC miniature black-light (UV) traps (model 1212; John W. Hock Company) baited with CO_2_ (Yeast; *Saccharomyces cerevisiae*). Traps were placed in two transects of 100 meters, separated 50 meters from each other (four traps per transect), with a distance of 30 meters between traps on the same transect to avoid competition between them; traps were placed at 60 cm from the ground. Traps were left active three hours during the morning (starting one hour before sunrise), at noon (from 12:30 to 15:30), and during late afternoon (one hour before sunset), for a total of nine hours of sampling per day; each site was sampled for two days each season. We used information provided by a global position system (Garmin GPSMAP 60) to determine the exact time of sunrise and sunset. Collected mosquitoes were sacrificed with chloroform gas, and then preserved in Petri dishes prepared with wax paper and cotton wool. Specimens were examined under a stereoscopic microscope and mounted on paper triangles held with entomological pins. We used different taxonomic publications for the identification of mosquito genera and species [25–34], reviewing the female external as well as male external and genitalia characteristics for identification. The identified mosquitoes were deposited into the entomological collection of the Institute of Ecology AC (IEXA, key 048.0198).

### Statistical analysis

Species accumulation curves and the Abundance-based Coverage Estimator (ACE) and Chao1 were used to determine sample efficiency [35–39]. This procedure was performed with EstimateS 8.2.0 [40].

Generalized additive mixed models (GAMM) were used to describe the spatiotemporal trends of both mosquito species richness and abundance. This statistical procedure is an extension of a generalized additive model which relaxes the assumption that the data are independent observations; hence, allowing for correlates, in our case each CDC miniature black-light (UV) trap [41]. An advantage of using GAMM to describe the abundance and richness at all studied seasons of the year and at the different sampling sites is that we can estimate the underlying trends of the data, without assuming that the trend has any specific functional form. Analyses were performed in R 2.15.3 (R Development Core Team 2013), using the following packages: mgcv [42], nlme [43], lme4 [44], gamm4 [42], and lattice [45].

Rank abundance curves were used to detect changes in the spatial and temporal structure of the mosquito assemblage [37]. ANCOVAs were used to assess significant differences in the slope between abundance curves (comparing seasons and sites). Values were log10 transformed because mosquito species abundances differed widely among sampling sites, seasons, and species. Subsequently, ANOVA tests were used to identify the effect that sites and seasons had on mosquito abundances. This analysis has been used primarily to analyze bird communities; this is the first time it is used in mosquito diversity studies [46].

To evaluate species composition between sites and seasons multivariate cluster analyses using βsim and the Morisita-Horn index were performed [37]. On the one hand, βsim quantifies the relative magnitude of won and lost species relative to the sample with fewer unique species [47]. Therefore, βsim allows the identification of changes in the composition of species; in this case for each land-use type and season. Because this is an analysis of dissimilarity we take 1-βsim. On the other hand, to analyze composition between sites and seasons using relative abundances, we performed a cluster analysis with the Morisita-Horn index that is a widely used species turnover index. It measures the probability that two randomly selected individuals, each from a different site or group, are of the same species [37]. The analysis and construction of the two clusters was performed in R v.3.0.2; the vegan and betadiver packages were used for the βsim index and the vegdist for the Morisita-Horn index respectively.

## Results

### Mosquito assemblage description

A total of 2536 adult mosquitoes belonging to 9 genera and 10 species, captured during the dry, rainy, and cold seasons of 2014 were processed. All 10 species were recorded at the five studied conditions. The ACE index estimates 10 species, whereas the Chao1 index estimates 9 species, suggesting that 100 % of the species were obtained in the present study (Additional file 1). The dominant species were *Aedes quadrivittatus* (36.16 %), *Wyeomyia adelpha* (37.78 %), *Wy. arthrostigma* (16.88 %), and *Culex restuans* (7.73 %). Among the rare species we found *Coquillettidia perturbans*, *Anopheles eiseni*, *Uranotaenia geometrica*, *Mansonia titillans* and *Sabethes gymnothorax*, which together represent 1.46 % of the sample (Table 1). Specimens of *Aedes* (*Ochlerotatus*) genus were not determined to the species level because only females were captured and male genitalia are required for species determination, but they were included in the study. The highest richness was recorded during the dry season (9 spp.), followed by the cold (8 spp.) and rainy seasons (7 spp.). In contrast, abundance was highest during the rainy season (1345 specimens), followed by the dry (671 specimens) and the cold seasons (520 specimens) (Table 1).

**Table 1:** Mosquito species numbers captured at each land-use type and seasons in the study area. Species codes on the first column are used in other tables and figures

^a^C	Species	Dry					Rainy					Cold					TOTAL
		CF	PF	UF	CS	PS	CF	PF	UF	CS	PS	CF	PF	UF	CS	PS	
Aq	*Aedes* (*Howardina*) *quadrivittatus* (Coquillett, 1902)	163	15	1	48	4	374	11	4	85	1	204	1	1	3	2	917
Cr	*Culex restuans* (Theobald, 1901)	8	35	73	5	3	11	14	24	8	2	1	0	7	2	3	196
Wa	*Wyeomyia adelpha* (Dyar and Knab, 1906)	30	24	31	2	8	205	215	13	147	10	213	42	1	13	4	958
Wt	*Wyeomyia arthrostigma* (Lutz, 1905)	17	18	160	1	3	0	83	121	7	6	2	2	7	0	1	428
A	*Aedes* (*Ochlerotatus*) sp.	0	0	9	0	0	0	0	0	0	0	0	2	0	0	0	11
Ug	*Uranotaenia geometrica* (Theobald, 1901)	1	4	0	0	1	0	0	0	1	0	0	0	0	1	6	14
Cp	*Coquillettidia perturbans* (Walker, 1856)	0	1	3	0	0	0	0	1	0	0	0	0	0	0	1	6
Ae	*Anopheles eiseni* (Coquillett, 1902)	2	0	0	0	0	0	0	0	0	0	0	0	0	1	0	3
Mt	*Mansonia titillans* (Walker, 1848)	0	0	0	0	0	0	0	2	0	0	0	0	0	0	0	2
Sg	*Sabethes gymnothorax* (Harbach and Petersen, 1992)	0	0	1	0	0	0	0	0	0	0	0	0	0	0	0	1
	Abundance	221	97	278	56	19	590	323	165	248	19	420	47	16	20	17	2536
	Richness	6	6	7	4	5	3	4	6	5	4	4	4	4	5	6	10

^a^ *CF* well-preserved montane cloud forest, *PF* peri-urban forest, *UF* urban forest, *CS* shade coffee plantation, *PS* cattle field, *C* species code

### Richness analysis

Significant effects of both seasons of the year and land-use type on species richness were observed (Table 2). The highest richness was found during the dry season (Table 2). For land use types, the well-preserved cloud forest (CF) and the shade coffee plantation (CS) had significantly lower species numbers in comparison to the urban forest (UF) (Table 2). UF had the highest richness (8 spp.) as compared with all other sites, which had 6 species each. Significant interaction effects between seasons and land-use types were detected, where CS and the peri-urban forest (PF) had higher species richness during the rainy season and UF had highest richness during both rainy and dry seasons (Table 2).

**Table 2:** General additive mixed model (GAMM) results using a Poisson distribution model. Significant *P* values are in boldface and non-significant trends in italics

	Abundance				Richness			
Components	Estimate	SE	*t*-value	*P*-value	Estimate	SE	*t*-value	*P*-value
Intercepts	3.918	0.243	16.107	**0.000**	0.798	0.187	4.263	**0.000**
Rainy	0.340	0.235	1.284	0.202	0.154	0.235	0.656	0.513
Dry	−0.642	0.221	−1.863	*0.065*	0.442	0.221	2.000	**0.048**
Peri-urban Forest (PF)	−2.190	0.273	−3.433	**0.001**	−0.406	0.273	−1.488	0.140
Urban Forest (UF)	−3.268	0.288	−3.093	**0.003**	−0.588	0.288	−2.038	**0.044**
Coffe Plantation (CS)	−3.045	0.311	−3.207	**0.002**	−0.811	0.311	−2.610	**0.010**
Cattle Field (PS)	−3.207	0.280	−3.126	**0.002**	−0.493	0.280	−1.760	*0.081*
Rainy:PF	1.588	0.347	2.270	**0.025**	0.619	0.347	1.785	*0.077*
Dry:PF	1.367	1.785	1.680	*0.096*	0.406	0.335	1.209	0.229
Rainy:UF	1.994	0.335	1.783	*0.077*	0.721	0.362	1.994	**0.049**
Dry:UF	3.497	0.362	3.121	**0.002**	0.690	0.346	1.995	**0.049**
Rainy:CS	2.178	0.346	2.178	**0.032**	0.902	0.381	2.366	**0.020**
Dry:CS	1.672	0.381	1.474	0.143	0.251	0.386	0.651	0.517
Rainy:PS	−0.229	0.386	−0.162	0.871	−0.355	0.404	−0.878	0.382
Dry:PS	0.753	0.404	0.528	0.599	−0.442	0.382	−1.156	0.250

### Abundance analysis

Significant effects of land-use type on species abundances were detected, but only a non-significant trend of seasonality during the dry season (Table 2). All sites (PF, UF, CS and cattle field (PS)) show statistically significant lower mosquito abundances in comparison to CF (Table 2). CF had a total of 1231 individuals, followed by PF (467), UF (459), CS (321), and PS (55). There were significant positive interaction effects between season and land-use type on mosquito abundances; higher abundances were recorded at PF and CS during the rainy season, and at UF during the dry season (Table 2).

### Assemblage structure

**Fig. 2:**
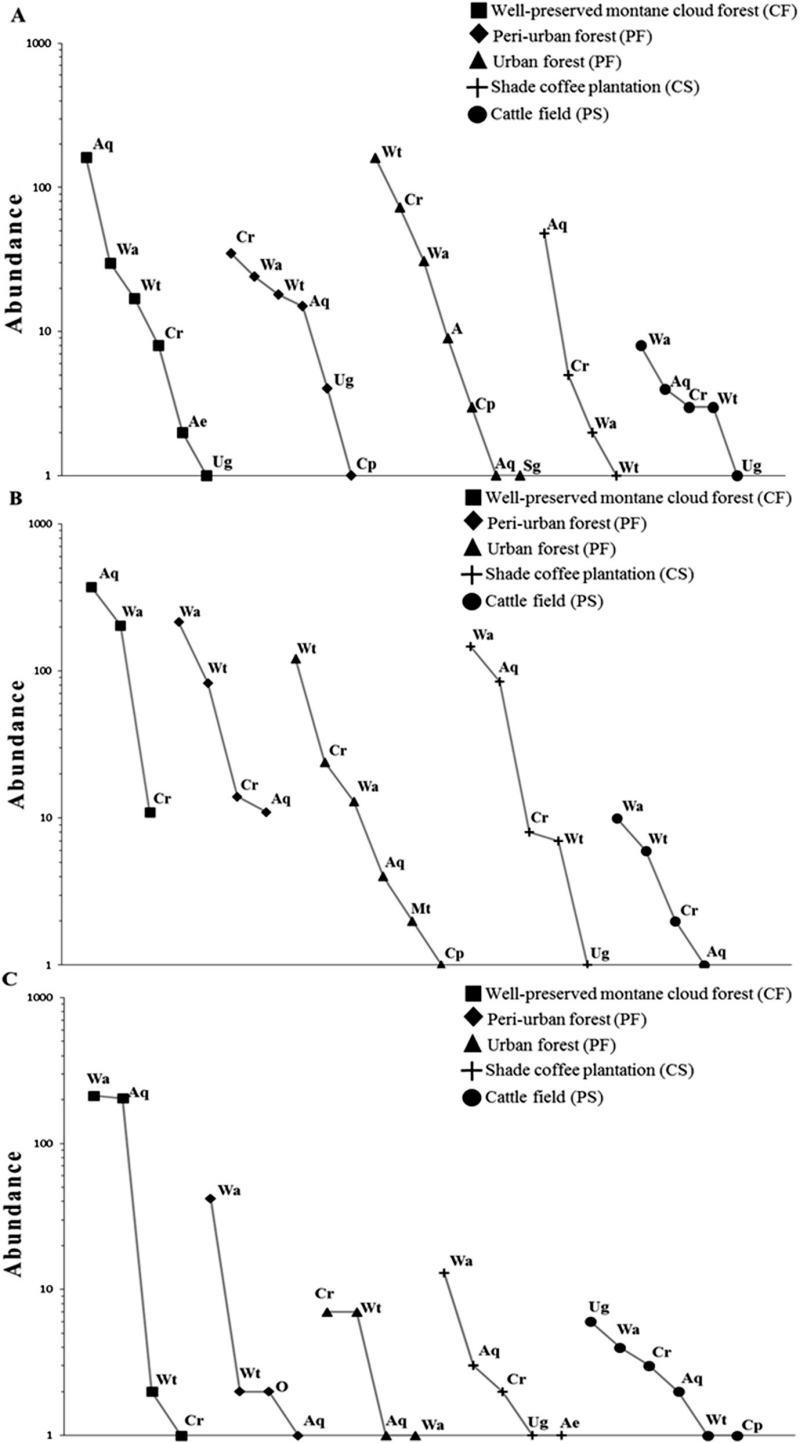
Rank-abundance curves of mosquito species captured at each site (well-preserved montane cloud forest (CF), peri-urban forest (PF), urban forest (UF), shade coffee plantation (CS) and cattle field (PS)) and seasons: **a**) dry, **b**) rainy, and **c**) cold. Species codes are shown in Table 1

In general, there is a higher evenness during the dry season in comparison to the other two seasons. Moreover, whereas CF, PF, and UF have the highest species richness across the year, CS and PS have the lowest (Fig. 2). The highest mosquito numbers and strong community dominance were observed during the rainy season due to the high abundances of *Aedes quadrivittatus* and *Wyeomyia adelpha* (Fig. 2), such dominance is reflected in the lowest species richness during this season. Strong community dominance during the cold season by the same two mosquito species mentioned above was also detected (Fig. 2). Furthermore, during the cold season PS presented the highest species richness. Following, detailed results for each season are provided.

During the dry season statistical significant differences in the mosquito assemblage structure were detected between the following pairs of land uses: CF- PF regarding abundance, PF-UF and PF- CS regarding abundance and richness (Table 3, Fig. 2). Rank-abundance curves showed that the urban forest (UF) is the site with the highest number of species and individuals (7 spp., 278 individuals), followed by the well-preserved montane cloud forest (CF) (6 spp., 221 individuals), the peri-urban forest (PF) (6 spp., 97 individuals), cattle field (PS) (5 spp., 19 individuals) and shade coffee plantation (CS) (4 spp., 56 individuals; Fig. 2). Four species were present in the five sites: *Aedes quadrivittatus*, *Culex restuans*, *Wyeomyia adelpha* and *Wy. arthrostigma,* with different abundance hierarchy. *Uranotaenia geometrica* was present only in three sites (CF, PF, and CS), *Coquillettidia perturbans* only in PF and UF, and *Sabethes gymnothorax* in UF (Fig. 2).

**Table 3:** Covariance analyses (ANCOVAs) comparing slopes of rank-abundance curves among land-use types and seasons. Significant *P* values are in boldface

**Dry**				
	^**a**^ **CF**	**PF**	**UF**	**CS**
**CF**	…	…	…	…
**PF**	**F**_**1, 35**_ **= 0.720;** ***P*** **= 3.62e-07**	…	…	…
**UF**	F_1, 22_ = 0.034; *P* = 0.249	**F**_**1, 25**_ **= 0.247;** ***P*** **= 4.364e-06**	…	…
**CS**	F_1, 21_ = 0.089; *P* = 0.089	**F**_**1, 24**_ **= 0.311;** ***P*** **= 9.758e-06**	F_1, 11_ = 0.013; *P* = 0.307	…
**PS**	F_1, 18_ = 0.002; *P* = 0.782	F_1, 21_ = 0.003; *P* = 0.462	F_1, 9_ = 0.011; *P* = 0.160	F_1, 7_ = 0.026; *P* =0.208
**Rainy**				
	**CF**	**PF**	**UF**	**CS**
**CF**	…	…	…	…
**PF**	**F**_**1, 35**_ **= 0.541;** ***P*** **= 8.433e-09**	…	…	…
**UF**	**F**_**1, 33**_ **= 0.364;** ***P*** **= 8.203e-06**	F_1, 36_ = 0.009; *P* = 0.393	…	…
**CS**	F_1, 30_ = 0.016; *P* = 0.151	**F**_**1, 33**_ **= 0.265;** ***P*** **= 1.310e-06**	**F**_**1, 31**_ **= 0.169;** ***P*** **= 0.0005**	…
**PS**	**F**_**1, 17**_ **= 0.074;** ***P*** **= 0.013**	**F**_**1, 20**_ **= 0.110;** ***P*** **= 0.002**	**F**_**1, 18**_ **= 0.105;** ***P*** **= 0.018**	**F**_**1, 15**_ **= 0.081;** ***P*** **= 0.001**
**Cold**				
	**CF**	**PF**	**UF**	**CS**
**CF**	…	…	…	…
**PF**	F_1, 18_ = 0.001; *P* = 0.692	…	…	…
**UF**	**F**_**1, 16**_ **= 0.083;** ***P*** **= 0.009**	**F**_**1, 8**_ **= 0.075;** ***P*** **= 0.013**	…	…
**CS**	**F**_**1, 13**_ **= 0.210;** ***P*** **= 0.0007**	**F**_**1, 5**_ **= 0.202;** ***P*** **= 0.006**	**F**_**1, 3**_ **= 0.262;** ***P*** **= 0.004**	…
**PS**	F_1, 15_ = 0.016; *P* = 0.214	F_1, 7_ = 0.018; *P* = 0.168	F_1, 5_ = 0.004; *P* = 0.340	**F**_**1, 2**_ **= 0.227;** ***P*** **= 0.013**

^a^ *CF* well-preserved montane cloud forest, *PF* peri-urban forest, *UF* urban forest, *CS* shade coffee plantation, *PS* = cattle field

Rank-abundance curves during the rainy season had the following general features: CF (3 spp., 590 individuals), UF (6 spp., 165 individuals), CS (5 spp., 248 individuals), PF (4 spp., 323 individuals), and PS (4 spp., 19 individuals, Fig. 2). There were significant differences in the mosquito assemblage regarding abundance and richness between CF-PF, CF-UF, CF-PS, PF-CS, UF-CS, UF-PS and CS-PS (Table 3). For the PF-PS pair, the obtained difference was only in reference to abundance (Table 3). Three species were present at all land-use types: *Aedes quadrivittatus*, *Culex restuans* and *Wyeomyia adelpha*, but with different abundances. *Wyeomyia arthrostigma* was not present in CF; *Cq. perturbans* and *Mansonia titillans* were found only in UF, whereas *Ur. geometrica* was found exclusively in CS (Fig. 2).

Rank-abundance curves during the cold season had the following general features: CF (4 spp., 420 individuals), PS (6 spp., 17 individuals), CS (5 spp., 20 individuals), PF (4 spp., 47 individuals), and UF (4 spp., 16 individuals), it is important to notice the large difference in total abundance between the CF and the other sites during this season. There were significant differences in the mosquito assemblage when considering both species richness and abundance between CF-UF, CF-CS, PF-US, UF-CS, and CS-PS (Table 3). *Aedes quadrivittatus* and *Wyeomyia adelpha* were present in all land-use types, *W. arthrostigma* was absent in C, and *Cx. restuans* was absent in PF. *Anopheles eiseni* was found exclusively in CS, *Cq. perturbans* in PS, and *Ur. geometrica* in CS and PS, In the UF, *Sabethes gymnothorax* was captured in the dry season and *Ma. titillans* was only collected in the rainy season (Fig. 2).

### Species composition

**Fig. 3:**
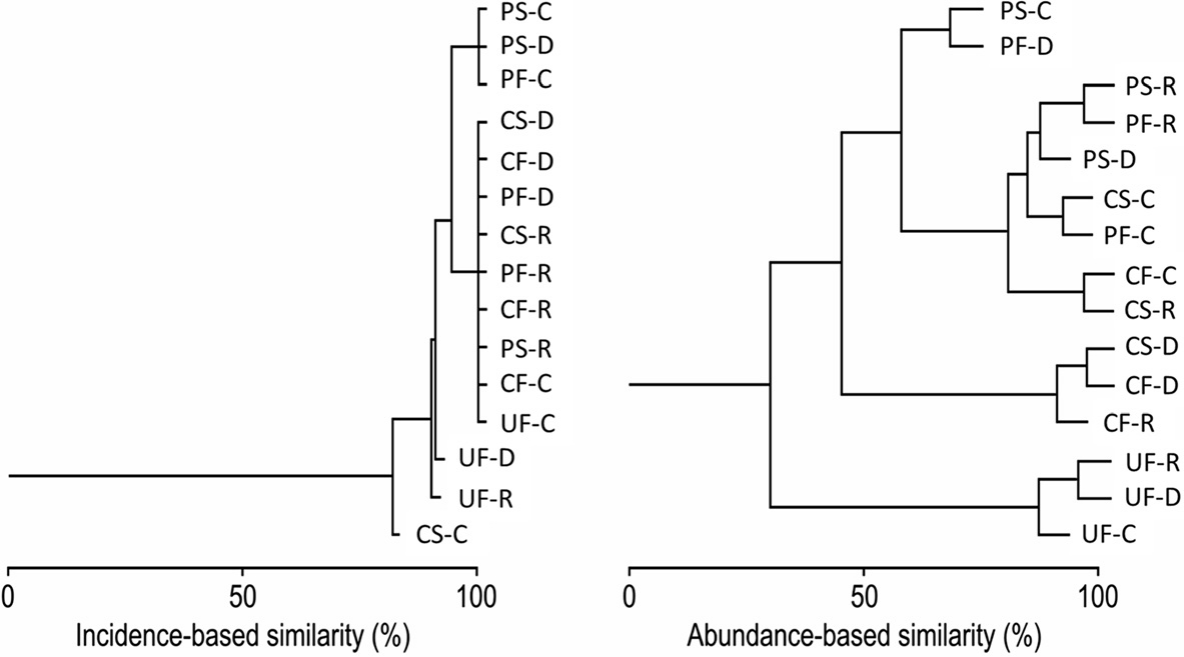
Dendrograms of cluster analyses showing similarities based on incidence (1-Β_sim_) and on species abundances (Morisita-Horn). Scales at the bottom of the figure represent similarity values (%). Sites: well-preserved montane cloud forest (CF), peri-urban forest (PF), urban forest (UF), shade coffee plantation (CS) and cattle field (PS); seasons: dry (D), rainy (R) and cold (C)

The cluster analysis for species incidences (1-β_sim_) shows that there is a high degree of similarity in species numbers across sites and seasons throughout the year, virtually the same mosquito species are found across the studied landscape (Fig. 3). Clusters integrated by CS, PS, and PF during the dry season, by CF, PF and PS during the rainy season, and by CF and UF during the cold season had 100 % species similarity (Fig. 3). The conditions with less similarity in species richness were CS during the cold season (83 %), and by UF during the dry and rainy seasons (>94 %, Fig. 3).

The cluster analysis based on the Morisita-Horn abundance index showed higher variation and lower similarity across sites and seasons in comparison to species composition (Fig. 3). There are four clusters with a high degree of similarity (>70 %) within themselves, but with low similarity (<35 %) among them (Fig. 3). We identified two clusters with similarity values above 90 %; one such cluster represented by UF in all seasons, indicating that this forest is very similar to itself across the year. *Aedes quadrivittatus*, *Culex restuans*, *Wyeomyia adelpha*, *Wy. arthrostigma* and *Coquillettidia perturbans* are the species that are driving this similarity pattern. The other cluster with high similarity includes CF during dry and rainy seasons, which are very similar to the shade coffee plantation (CS) during the dry season. The well-preserved cloud forest (CF) mosquito species abundances change during the cold season, making it more similar to CS during the rainy season. *Ae. quadrivittatus*, *Cx. restuans*, *Wy. adelpha*, *Wy. arthrostigma*, *Uranotaenia geometrica* and *Anopheles eiseni* are the species that are driving the similarities between CF and CS. The peri-urban forest (PF) is very similar to CS during the cold season and to PS during the rainy season (Fig. 3). The species driving the similarities among such sites are *Ae. quadrivittatus*, *Cx. restuans*, *Wy. adelpha* and *Wy. arthrostigma*.

## Discussion

This study focused on understanding responses of a mosquito (Diptera: Culicidae) assemblage to different land-use types throughout the year, within a landscape originally dominated by neotropical montane cloud forest. In general, our results show that the region, regardless of land-use type, is dominated by four species (*Aedes quadrivittatus*, *Wyeomyia adelpha*, *Wy. arthrostigma*, and *Culex restuans*), and that communities are very similar in terms of species richness: more than half of the species are shared among the five conditions during the three seasons of the year. In terms of abundances, mosquito communities show higher variability and lower similarity among land-use types and seasons, indicating that resources for mosquito development vary across space and time. In particular, the hierarchy of dominant species changes in reference to land-use type and sometimes across seasons within the same habitat type. Wooded areas (e.g., well-preserved montane cloud forests, secondary cloud forests, shade coffee plantations) can account for the similarity of mosquito communities across sites; even cattle ranches in the area are small, with interspersed trees, and surrounded by forests. Furthermore, flying habits of mosquito species (e.g., *Ma. titillans* and *Cq. perturbans*, which can fly several miles from their oviposition sites [28, 47]) can also be responsible for the high species similarity observed. Another important feature is the presence of epiphytes, such as bromeliads across the landscape, which are commonly used by species such as *Ae. quadrivittatus* and *Wy. adelpha* for oviposition [27, 38]. Hence, the mosquito assemblage is highly homogeneous across this local landscape, which can be the result of high connectivity provided by a largely forested matrix.

Mosquito species numbers and abundances are known to readily respond to abiotic conditions [14, 16]. Low temperatures during the cold months of the year are most likely directly affecting mosquito richness and abundance because low temperatures reduce mosquito breeding and feeding activities [48-50]. An interesting aspect is the high composition similarity of the urban forest (UF) throughout the year for both species richness and abundance. Being surrounded by unsuitable habitat (gray areas of xcities) for many mosquito species, the assemblage is isolated from other nearby sites in the landscape, which reduces the possibility of dispersal and may explain the relative structural difference in the mosquito assemblage in comparison to the other sampled conditions (e.g., the singleton *Sa. gymnothorax*, Fig. 3). In contrast, the well-preserved montane cloud forest and the shade coffee plantation are similar in mosquito species. This can be explained by the proximity of both sites as many species of mosquitoes can travel several kilometers to feed in these environments, such as the dominant species *Ae. quadrivittatus* and *Wy. adelpha*.

Richness and abundance analyses showed that there is an increase in richness for those environments close to or at the city (i.e., urban and peri-urban forests), but abundance is higher at the well-preserved montane cloud forest. Richness and abundance do not always decrease progressively or vary in parallel with increasing habitat modification [51, 52]. Forest disturbance apparently cause an increase in species richness; as an example, some studies have shown that species richness of dung beetles is higher in logged forests compared to primary forests [53]. Another example is the increase in numbers of nesting bees and wasps as land-use intensity increases [54]. Secondary forests and agroforestry systems probably maintain diversity [51]. Hence, it is necessary to delve further in the relevance of human dominated habitats, in particular urban ones that will be more common in the near future, for both to understand how they help protect biodiversity and also how they disrupt host-parasite interactions in such a way that wildlife pathogens can become a health issue [55–57].

The urban environment may have a negative influence on biodiversity due to the generated isolation by gray areas (buildings and roads), pollution, and the use of insecticides [58]. In such conditions, only mosquito species that can adapt will increase their abundances. For example, females of *Ae. quadrivittatus* are attracted to humans; they are able to feed throughout the day, and viruses have been isolated from this species in Panama [59]*. Wy. arthrostigma* is the most abundant species in the urban forest, this may be due to the ability of this species to use holes in trees and hollow shafts for oviposition [60], favored by the presence of bamboo patches, which is one of the preferred oviposition sites. In the case of *Cx. restuans*, the larvae develop in a wide xvariety of water sources such as ditches, pools, streams, forests, and artificial reservoirs (e.g., tires, cans, fountains). This species reaches its highest abundance during the spring and early summer throughout most of its range, and occurs in fewer numbers during late summer and autumn [29, 61]. At our study sites, this species had higher abundance in the urban forest. Several studies have demonstrated the ability of *Cx. restuans* to transmit avian malaria [62–65], which can represent a health risk for native birds using these forest remnants within cities. Furthermore, among the species of medical importance are *Mansonia titillans*, which is vector of Venezuelan equine encephalitis virus (VEE), it was the primary vector for the 1942–1943 epidemic in Trinidad. This species is known to be a vector of filarid nematodes as well [29]. The females of *Coquillettidia perturbans* bite mainly at night, usually more active during the first hours of the night. Occasionally, it bites humans during the day inside houses and is a vector of eastern equine encephalitis (EEE) [66]. The latter two species are rare at our study sites, but the individuals we collected were mainly from the urban forest. Hence, it is likely that humans and other wild vertebrates using this type of environment will be at higher risk of infection by the above-mentioned parasite groups, in particular considering that wild host populations (i.e., urban avoiders restricted to green areas of cities) are packed within relatively small areas.

Some mosquito species exhibit a high degree of specialization in their host and oviposition site selection, while others are completely generalist and opportunistic [67–69]. Changes in host abundances due to anthropogenic impacts can affect both host and habitat choice, especially if blood-sucking species are generalists and mobile (e.g., [15, 16, 70]). The peri-urban forest and the cattle field had the same mosquito assemblage structure during the rainy season, but with different abundances; forest fragments and urban areas are bordering the cattle field, providing similar anthropogenic influences for both sites. It is possible that mosquitoes use the cattle field as a feeding site because it provides food all year round due to the presence of domestic animals, such as cows and chickens; at the same time, it provides shelter because of the presence of nearby forest fragments and secondary vegetation [70]. Hence, it is necessary that future studies consider sampling a buffer of the surrounding habitat to the focal one, in order to have a better understanding of mosquito community spatial and temporal dynamics.

Analyzing richness and abundance of mosquito assemblages separately can be a better strategy to identify differences under similar environmental conditions in comparison to the use of diversity indexes. Land-use change and habitat alteration have consequences in mosquito vector assemblage structure. Mosquito diversity studies, as the one presented here, represent an important first step for planning urban development in terms of sanitarian strategies for vector-borne disease prevention, as well as developing surveillance programs to prevent zoonotic disease. Finally, mosquito community studies can be useful to monitor ecosystem health [71].

## Conclusions

This study showed that land-use changes and seasonality influence mosquito community structure, in particular in terms of species abundances. However, our results also demonstrate that the mosquito assemblage is rather homogeneous (i.e., highly similar richness) across the studied landscape, suggesting a high degree of spatial connectivity, even when land-use types represent a drastic change (e.g., cattle ranches) compared to the original cloud forest. Near the city environments mosquito richness is greater than at the well-preserved cloud forest, but the later has higher abundance compared to all other sites. The highest species richness was recorded during the dry season, but the highest abundance was recorded during the rainy season. Finally, our work generates important information to understand mosquito diversity in reference to land-use changes within landscapes, which is a potentially useful tool in the development of urban planning and surveillance programs focused mainly on mosquito species of medical and veterinary importance.

## Additional file

Additional file 1:
**Species accumulation curve to assess sampling effort, showing that 100 % of the species were obtained in the present study.** (PNG 89 kb)

## Abbreviations

CF: Well-preserved montane cloud forest
PF: Peri-urban forest
UF: Urban forest
CS: Shade coffee plantation
PS: Cattle field
VEE: Venezuelan equine encephalitis virus
EEE: Eastern equine encephalitis
ACE: Abundance-based coverage estimator
GAMM: Generalized additive mixed models
