# Supplementary figures and images for: Spatiotemporal variation of mosquito diversity (Diptera: Culicidae) at places with different land-use types within a neotropical montane cloud forest matrix
Source: Parasit Vectors. 2015 Sep 24;8:487. doi: 10.1186/s13071-015-1086-9 (PMC4581103; doi:10.1186/s13071-015-1086-9)

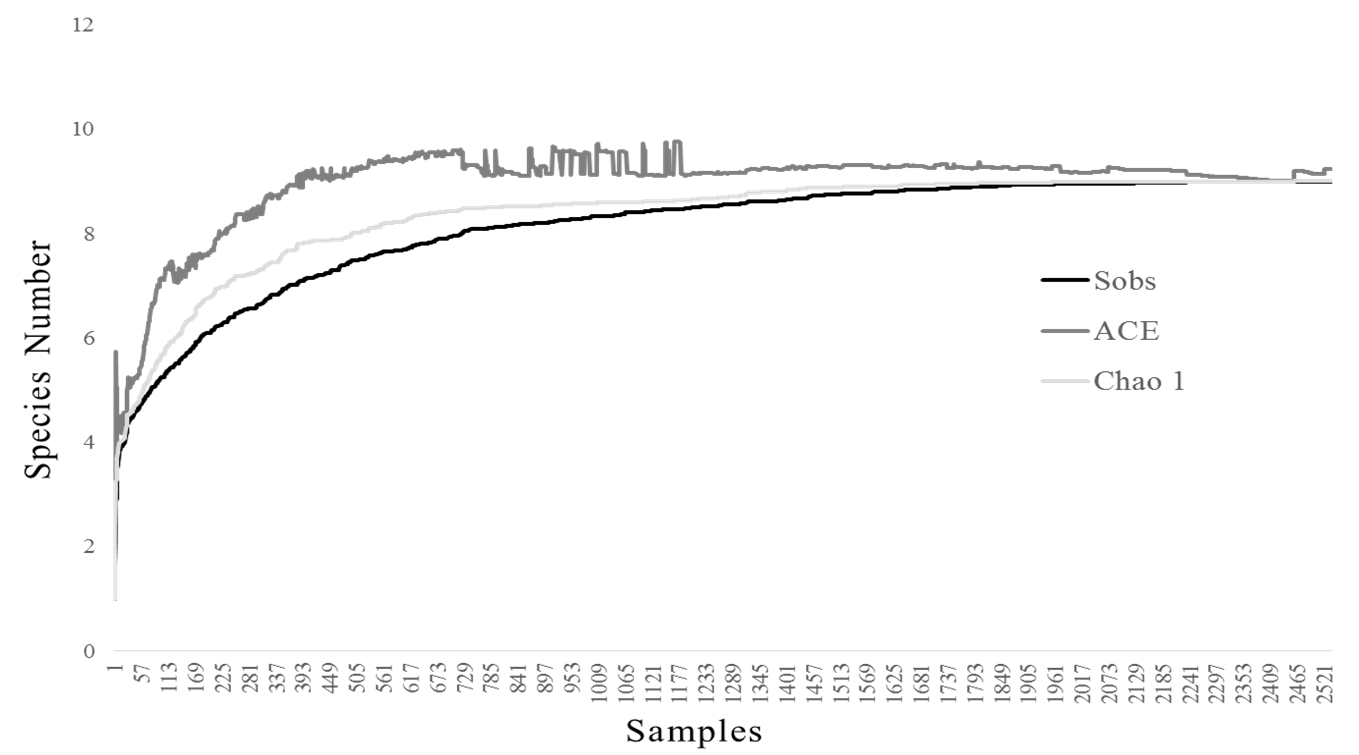

Supplement: Additional file 1: — Species accumulation curve to assess sampling effort, showing that 100 % of the species were obtained in the present study. (PNG 89 kb) [file 13071_2015_1086_MOESM1_ESM.png]
